# Overall trend towards headache remission during the COVID-19 pandemic among Chinese patients with pre-existing headache highlights the role of family support

**DOI:** 10.1186/s12883-021-02216-6

**Published:** 2021-06-15

**Authors:** Qiaoyu Gong, Shuping Liu, Ruiting Li, Lihua Yao, Zheman Xiao

**Affiliations:** 1 Department of Neurology, Renmin Hospital of Wuhan University, 99 Zhang Zhidong Road, Wuchang District, Wuhan, Hubei Province 430060 P. R. China; 2 Department of Psychiatry, Renmin Hospital of Wuhan University, Wuhan, Hubei Province 430060 P. R. China

**Keywords:** COVID-19, Impact, Headache, Remission, Family support

## Abstract

**Background:**

The global status of the COVID-19 pandemic is not optimistic. This is a particularly vulnerable time for patients with pre-existing headache disorders. The present study aimed to investigate the impact of the COVID-19 pandemic on headache patients in China.

**Methods:**

A survey was conducted through an online survey platform on June 6, 2020. Demographic characteristics, the PHQ-9, the ISI, a COVID-19 questionnaire and a headache profile survey were included in the online questionnaire.

**Results:**

Eventually, a total of 15,000 participants from China completed the online questionnaire. Among them, 2806 participants had pre-existing headache disorders. Our analysis showed reductions in the duration of headaches (3.414 ± 6.859 vs 4.033 ± 7.325 h, *P*<0.001), number of headache days per month (1.788 ± 2.989 vs 2.092 ± 3.694, *P*<0.001), and headache intensity (4.110 ± 1.609 vs 4.290 ± 1.680, *P*<0.001) during the COVID-19 pandemic. Smoking (OR = 1.397, 95% CI 1.090 to 1.790, *P* = 0.008) and getting support from family members during social isolation (OR = 1.656, 95% CI 1.075 to 2.550, *P* = 0.022) were independent factors affecting the reduction in the duration of headaches. Education level (OR = 1.478, 95% CI 1.103 to 1.980, *P* = 0.009) and having a relative or acquaintance who contracted COVID-19 (OR = 0.643, 95% CI 0.458 to 0.902, *P* = 0.011) were the independent factors affecting the reduction in headache severity. Living in the Wuhan area, having symptoms or a diagnosis of COVID-19 and having relatives or acquaintances who had contracted COVID-19 were associated with the worsening of headaches.

**Conclusions:**

Participants experienced an overall trend towards the improvement of headaches during the COVID-19 pandemic. Family support might play an important role in the improvement of headaches.

**Supplementary Information:**

The online version contains supplementary material available at 10.1186/s12883-021-02216-6.

## Background

Coronavirus disease 2019 (COVID-19), which was first reported in Wuhan, China, has caused a global pandemic due to its rapid human-to-human transmission via respiratory droplets and direct contact [1, 2]. Currently, there have been more than 93 million confirmed COVID-19 cases and more than two million deaths worldwide. To minimize the risk of exposure and prevent the further spread of COVID-19, “social distancing” is advocated. The implementation of social distancing has disrupting our normal lives, work and medical care.

Headache is an important neurological symptom reported in COVID-19 patients [3]. Almost 11–34% of hospitalized COVID-19 patients present with headache [4]. More importantly, the sudden emergence of COVID-19 was a shock to the public and generated many psychological problems [5]. Headache patients, particularly those with migraine, are more vulnerable to such major stressful life events. Anxiety and stress caused by the COVID-19 pandemic may aggravate headaches. Additionally, the COVID-19 outbreak has drastically affected the medical care received by patients with pre-existing headache disorders [6].

The global status of the COVID-19 pandemic is not optimistic. This is a particularly vulnerable time for patients with pre-existing headache disorders and has led to widespread concern among headache specialists. It is imperative to understand the present status of headache patients during the COVID-19 pandemic. To our knowledge, no research has been reported on headache patients in China during the COVID-19 pandemic. Thus, the present study was performed to investigate the impact of the COVID-19 pandemic on headache patients in China and to further explore potential factors aggravating or relieving their headaches.

## Methods

### Design and participants

A survey was conducted through an online survey platform from 00:00 to 23:59 on June 6, 2020, which was completely voluntary and non-commercial. The anonymous questionnaire was designed by the research team from the Mental Health Centre of Renmin Hospital of Wuhan University and the School of Computing of Huazhong University of Science and Technology. All participants signed an informed consent form before completing the questionnaire and could withdraw from the survey at any time. All online questionnaires were in Chinese. People throughout China were encouraged to complete and submit the online questionnaires in a timely manner. Participants who competed all the items on the online questionnaire were included.

This study was approved by the Ethics Committee of Renmin Hospital of Wuhan University.

### Variables and instruments

#### Demographic characteristics

Demographic data included sex, age, education level, chronic disease history, smoking status and alcohol consumption status.

#### Depressive symptoms

The Patient Health Questionnaire-9 (PHQ-9), a nine-item questionnaire, was used to assess depressive symptoms. It is based on the criteria of the DSM-IV (the Diagnostic and Statistical Manual of Mental Disorders, Fourth Edition) and assesses each of the criteria of the DSM-IV for major depressive disorder. Items are rated on a 4-point Likert-type scale, ranging from 0 (not at all) to 3 (nearly every day). The total score can range from 0 to 27, with high scores indicating severe depression [7].

#### Sleep quality

The Insomnia Severity Index (ISI), a brief self-reported instrument, was used to assess subjective sleep quality. The ISI comprises seven items evaluating the severity of difficulties with sleep onset and sleep maintenance (waking both at night and in the early morning), satisfaction with current sleep patterns, interference with daily functioning, noticeable impairment attributed to sleep problems, and degree of distress or concern caused by sleep problems. Each item is rated on a 4-point Likert-type scale, ranging from 0 to 3, and the total score ranges from 0 to 28. A higher score suggests more severe insomnia [8].

#### COVID-19 questionnaire

The COVID-19 questionnaire included items to ascertain whether the respondent had experienced COVID-19 symptoms or received a diagnosis of COVID-19, had a relative or acquaintance who had contracted COVID-19, had received support from family members, and had received support from others during social isolation.

The Impact of Event Scale Revised (IES-R), which is a self-administered questionnaire, was used to assess the psychological impact of the COVID-19 pandemic. The IES-R includes three subscales and is designed to assess the mean levels of avoidance, intrusion, and hyperarousal. It comprises 22 items, which are scored on a 5-point scale, ranging from 0 to 4, and the total score ranges from 0 to 88. A higher score suggests more severe symptoms [5].

#### Headache profile

The headache profile section included items on a history of pre-existing headache disorders, the characteristics of the accompanying symptoms (nausea, vomiting, photophobia, phonophobia and osmophobia, aggravation by or causing avoidance of routine physical activity, lacrimation, rhinorrhoea, conjunctival injection, eye bilges and remission induced by massage), headache triggers (increased stress, short sleep duration, excessive sleep, menstruation, bright light, noise or special smell, special foods and alcohol), headache intensity, headache days per month, headache duration at baseline and during the COVID-19 pandemic, and factors associated with headache during the COVID-19 pandemic (stress, sleep quality, changes in lifestyles and decreased social interaction).

We asked the participants about their headache characteristics before the COVID-19 pandemic (3 months before the start of lockdown) and during the COVID-19 pandemic (3 months after the start of lockdown). Headache outcomes included remission, no change, and exacerbation. Participants who reported decreases in headache frequency, headache severity or headache duration were included in the remission group; those who reported no changes were included in the unchanged group; those who reported increases were included in the aggravation group.

### Statistical analysis

The statistical analysis was performed with SPSS v25.0 software (IBM, West Grove, Pennsylvania, USA). Categorical data are reported as proportions, while continuous data are reported as the means and standard deviations. The chi-square test with subsequent Bonferroni correction was used to determine whether differences in proportions were significant. The Mann-Whitney U test or the Wilcoxon signed-rank test was used to compare non-normally distributed data. Binary logistic regression was first performed to identify the variables associated with headache relief. Then, multivariate logistic regression analyses were performed to verify the independent factors influencing headache relief. The Hosmer-Lemeshow test was performed to estimate the fit of the overall model. A *p*-value < 0.05 was considered to be statistically significant.

## Results

### Demographic data

Eventually, a total of 15,000 participants from China completed the online questionnaire. Of these, 22 (0.13%) respondents had been diagnosed with COVID-19. Overall, the majority of respondents were female (8569/15000, 57.13%), aged 30–50 years (10,061/15000, 67.07), and well educated (11,417/15000, 76.11%), defined as having at least a bachelor’s degree. Among them, 2806 participants (18.71%) had pre-existing headache disorders. The demographic characteristics of all survey respondents are shown in Table 1.

**Table 1:** Demographic characteristics of all survey respondents (*n* = 15,000)

Characteristics	N (%)
**Age (years)**	
< 30	3726 (24.84)
30–50	10,061 (67.07)
> 50	1213 (8.09)
**Male**	6431 (42.87)
**Education level**	
junior colleague or lower	5885 (39.23)
University or above	9115 (60.77)
**Chronic disease history**	2126 (14.17)
**Smoking**	2140 (14.27)
**Drinking**	5585 (37.23)
**Living in the Wuhan area**	439 (29.27)
**Having symptoms or a diagnosis of COVID-19**	377 (2.51)
**Having relatives or acquaintances contracting COVID-19**	1130 (7.53)
**Getting support from family members**	
Unchanged or more	14,398 (95.99)
Less	602 (4.01)
**Getting support from the outside world**	
Unchanged or more	13,377 (89.18)
Less	1623 (10.82)
**ISI score**	
≤ 8	11,181 (74.54)
> 8	3819 (25.46)
**PHQ9 score**	
<4	8674 (57.83)
≥ 4	6326 (42.17)
**ISER score**	
≤ 23	11,480 (76.54)
24–32	1634 (10.89)
33–36	470 (3.13)
> 37	1416 (9.44)

### Epidemiological data

The prevalence of headache peaked during adolescence (under 18 years old), followed by middle age (31–40 years). Headache was more prevalent in females (20.57%) than in males (16.22%) (Additional file 1).

**Fig. 1:**
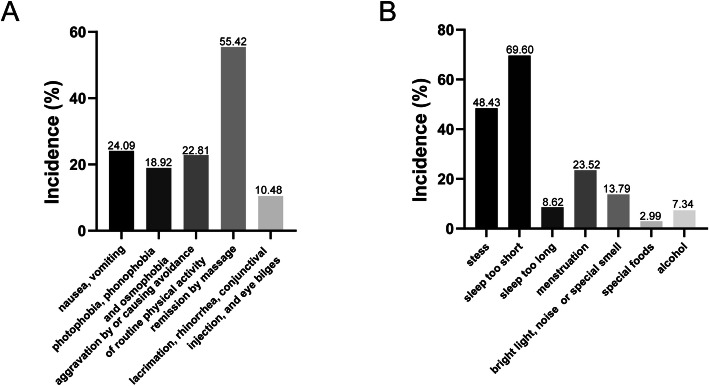
Characteristics of accompanying symptoms (**A**) and headache triggers (**B**)

A total of 676 of the 2806 headache participants (24.09%) reported accompanying nausea or vomiting; 531 (18.92%) experienced photophobia, phonophobia or osmophobia; 641 (18.92%) experienced aggravation by or causing avoidance of routine physical activity; 1555 (55.42%) experienced remission induced by massage; and 294 (10.48%) experienced lacrimation, rhinorrhoea, conjunctival injection or eye bilges. Participants reported that increased stress (48.43%), short sleep duration (69.60%), excessive sleep (8.62%), menstruation (23.52%), bright light, noises or specific smells (13.79%), specific foods (2.99%) and alcohol (7.34%) induced headache attacks (Fig. 1).

### Headache during COVID-19

Our analysis showed reductions in headache duration (3.414 ± 6.859 vs 4.033 ± 7.325 h, *P*<0.001), the number of headache days per month (1.788 ± 2.989 vs 2.092 ± 3.694, *P*<0.001), and headache intensity (4.110 ± 1.609 vs 4.290 ± 1.680, *P*<0.001) during the COVID-19 pandemic (Table 2).

**Table 2:** Comparisons of headache intensity, headache days per month, and headache duration at baseline and during the COVID-19 pandemic

variables	Before COVID-19 (*n* = 2806)	During COVID-19 (*n* = 2806)	*P* value
Headache severity	4.290 ± 1.680	4.110 ± 1.609	**< 0.001**
Headache duration (h)	4.033 ± 7.325	3.414 ± 6.859	**< 0.001**
Headache days per month	2.092 ± 3.694	1.788 ± 2.989	**< 0.001**

**Fig. 2:**
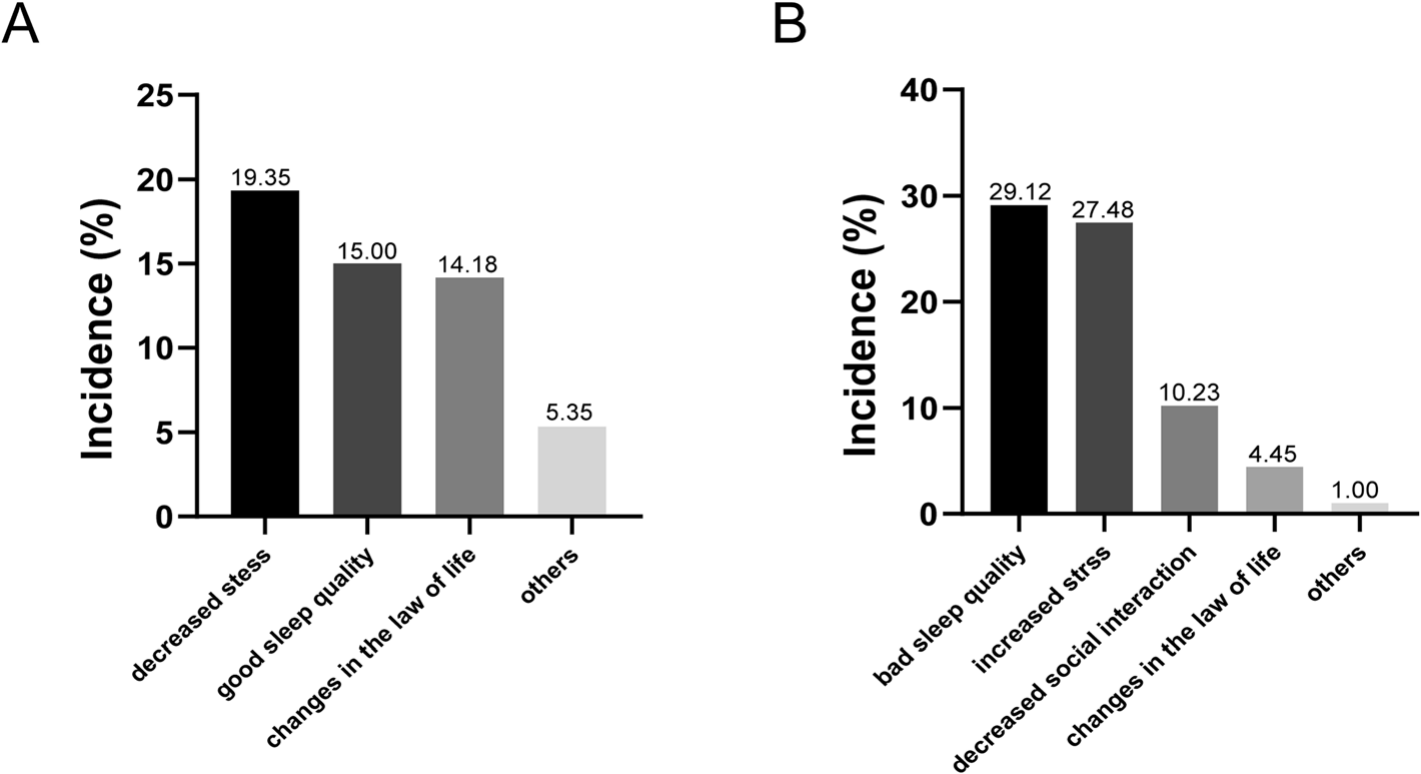
Self-reported factors associated with the remission of (**A**) or deterioration in (**B**) headache disorders during COVID-19

Participants reported decreased stress (19.35%), changes in lifestyles (14.18%), good sleep quality (15.00%) or others (5.35%) as factors associated with headache remission, while increased stress (27.48%), poor sleep quality (29.12%), decreased social interaction (10.23%), changes in lifestyles (4.45%) or others (1.00%) were factors associated with headache aggravation (Fig. 2).

**Fig. 3:**
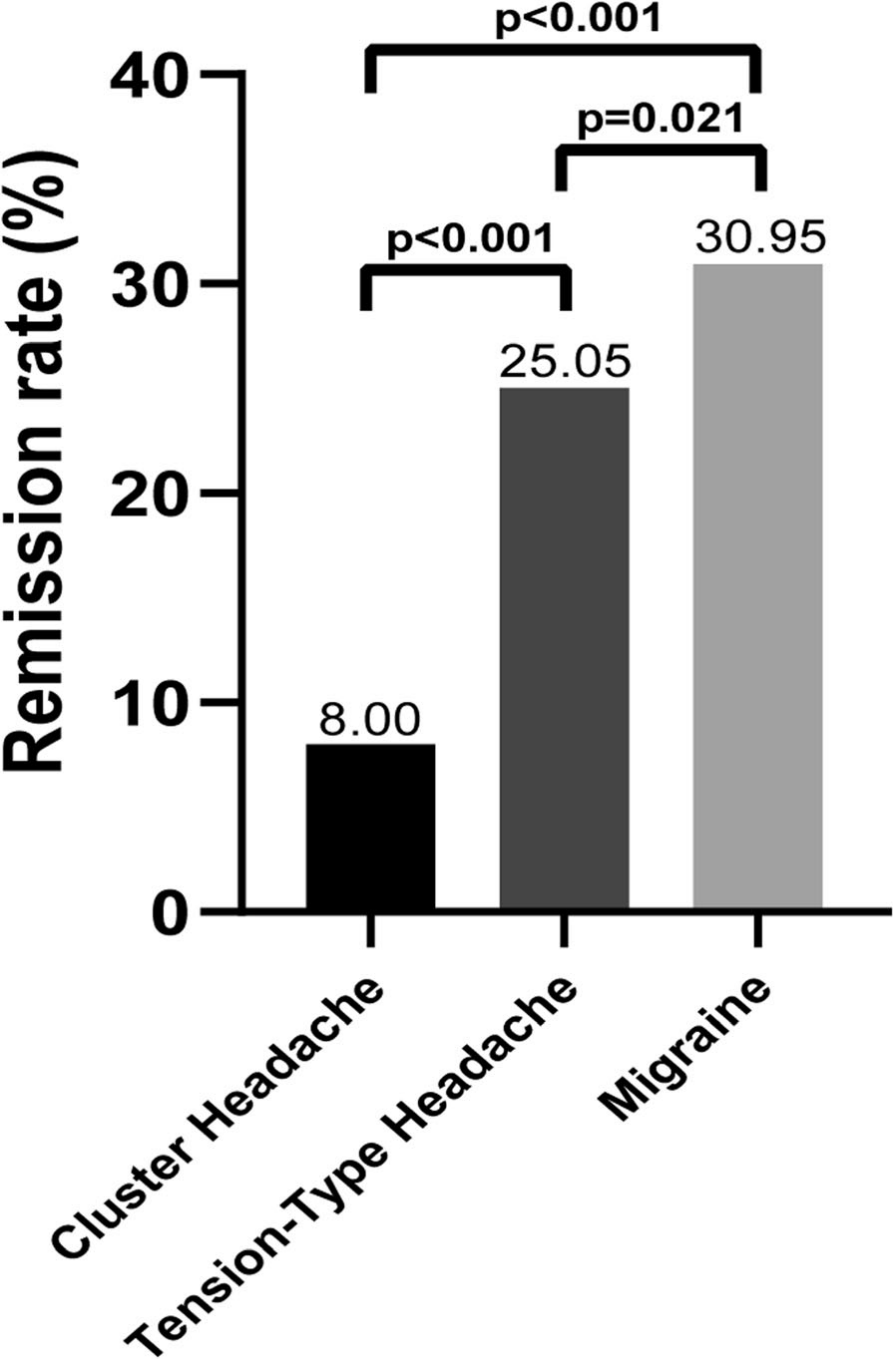
Proportions of participants who experienced a reduction in headache duration in the TTH, migraine and CH groups

Application of the International Headache Society (IHS) criteria [9] generated the following diagnoses: 378 respondents had migraine, 1385 had tension-type headache (TTH), and 100 had cluster headache (CH). A greater proportion of the participants with migraine experienced a reduction in headache duration than participants with TTH (30.95% vs 25.05%, *P* = 0.021) and CH (30.95% vs 8.00%, *P*<0.001). In addition, a greater proportion of the participants with TTH experienced a reduction in headache duration than participants with CH (25.05% vs 8.00%, *P*<0.001) (Fig. 3).

### Between-group differences

A total of 686 of the 2806 participants (24.45%) experienced reductions in headache duration during the COVID-19 pandemic, 1936 (68.99%) reported no changes in duration, and 184 (6.56%) reported longer headache durations. The aggravation group had significantly higher proportions of participants who smoked, lived in the Wuhan area, had a relative or acquaintance who contracted COVID-19, and received less support from family members during social isolation and had significantly higher ISI, PHQ-9 and ISE-R scores than the remission group (*P*<0.001, *P* = 0.009, *P* = 0.002, *P*<0.001, *P*<0.001, *P*<0.001, *P*<0.001, respectively) and the unchanged group (*P* = 0.005, *P* = 0.008, *P* = 0.002, *P* = 0.005, *P*<0.001, *P*<0.001, *P*<0.001, respectively). The aggravation group also had a significantly higher proportion of participants who had COVID-19 symptoms or a diagnosis of COVID-19 than the unchanged group (*P*<0.001) and a higher proportion of participants who received less support from those other than family members during social isolation than the remission group (*P* = 0.011) (Table 3).

**Table 3:** Comparison of demographic characteristics among the remission group, unchanged group and aggravation group according to headache duration

characteristics	The remission group^**1**^ (*n* = 686)	The unchanged group^**2**^ (*n* = 1936)	The aggravation group^**3**^ (*n* = 184)	*P* value	*P* value Post hoc		
					1 vs 2	1 vs 3	2 vs 3
**Male, n (%)**	251 (36.59)	715 (36.93)	77 (44.85)	0.393			
**Age, n (%)**				0.253			
< 30	176 (25.66)	441 (22.78)	53 (28.80)				
30–50	463 (67.49)	1371 (70.82)	120 (65.22)				
> 50	47 (6.85)	124 (6.40)	11 (5.98)				
**Education level, n (%)**				**0.035**	0.152	0.172	0.018
junior colleague or lower	150 (21.87)	374 (19.32)	49 (26.63)				
University or above	536 (78.13)	1562 (80.68)	135 (73.37)				
**Chronic disease history, n (%)**	167 (24.34)	444 (22.93)	52 (28.26)	0.253			
**Smoking, n (%)**	91 (13.27)	334 (17.25)	47 (25.54)	**< 0.001**	**0.015**	**< 0.001**	**0.005**
**Drinking, n (%)**	279 (40.67)	813 (41.99)	88 (47.83)	0.217			
**Living in the Wuhan area, n(%)**	20 (2.92)	63 (3.25)	13 (7.07)	**0.018**	0.663	**0.009**	**0.008**
**Having symptoms or a diagnosis of COVID-19, n(%)**	45 (6.56)	86 (4.44)	20 (10.87)	**< 0.001**	0.029	0.048	**< 0.001**
**Having relatives or acquaintances contracting COVID-19, n (%)**	80 (11.66)	240 (12.40)	38 (20.65)	**0.004**	0.613	**0.002**	**0.002**
**Getting support from family members, n (%)**				**0.001**	**0.015**	**< 0.001**	**0.005**
Unchanged or more	659 (96.06)	1811 (93.54)	163 (88.59)				
Less	27 (3.94)	125 (6.46)	21 (11.41)				
**Getting support from the outside world, n (%)**				**0.028**	0.056	**0.011**	0.120
Unchanged or more	599 (87.32)	1632 (84.30)	147 (79.89)				
Less	87 (12.68)	304 (15.70)	37 (20.11)				
**ISI score**	6.70 ± 5.30	8.16 ± 5.67	10.22 ± 6.01	**< 0.001**	**< 0.001**	**< 0.001**	**< 0.001**
**PHQ9 score**	5.99 ± 4.74	6.92 ± 4.93	8.74 ± 4.89	**< 0.001**	**< 0.001**	**< 0.001**	**< 0.001**
**ISER score**	17.99 ± 14.18	21.08 ± 14.63	27.71 ± 16.96	**< 0.001**	**< 0.001**	**< 0.001**	**< 0.001**

A total of 737 of the 2806 participants (26.27%) experienced improvements in headache frequency (days per month). During the COVID-19 pandemic, 1790 (63.79%) participants reported that their headache frequency did not change, and 279 (9.94%) reported that it had increased. The aggravation group had significantly higher proportions of participants who had underlying diseases, lived in the Wuhan area, had COVID-19 symptoms or a diagnosis of COVID-19, and had a relative or acquaintance who contracted COVID-19 than the remission group (*P* = 0.005, *P* = 0.001, *P*<0.001, *P*<0.001, *P*<0.001, respectively) and the unchanged group (*P*<0.001, *P* = 0.004, *P*<0.001, *P*<0.001, respectively). The aggravation group also had significantly higher proportions of participants who were male (*P* = 0.010), drank (*P* = 0.003) and received less support from family members during social isolation (*P* = 0.007) than the unchanged group and had higher ISI (*P*<0.001), PHQ-9 (*P* = 0.006) and ISE-R (*P* = 0.002) scores than the remission group (Table 4).

**Table 4:** Comparison of demographic characteristics among the remission group, unchanged group and aggravation group according to headache frequency

characteristics	The remission group^**1**^ (*n* = 737)	The unchanged group^**2**^ (*n* = 1790)	The aggravation group^**3**^ (*n* = 279)	*P* value	*P* value Post hoc		
					1 vs 2	1 vs 3	2 vs 3
**Male, n (%)**	289 (39.21)	633 (35.36)	21 (43.47)	**0.015**	0.068	0.228	**0.010**
**Age, n (%)**				0.431			
< 30	185 (25.10)	410 (22.91)	75 (26.88)				
30–50	502 (68.11)	1263 (70.56)	190 (68.10)				
> 50	50 (6.79)	117 (6.53)	14 (7.82)				
**Education level, n (%)**				0.260			
junior colleague or lower	152 (20.62)	354 (19.78)	64 (24.01)				
University or above	585 (79.38)	1456 (80.22)	212 (75.99)				
**Chronic disease history, n (%)**	184 (24.97)	385 (21.51)	94 (33.69)	**< 0.001**	0.059	**0.005**	**< 0.001**
**Smoking, n (%)**	123 (16.69)	291 (16.26)	58 (20.79)	0.169			
**Drinking, n (%)**	316 (42.88)	725 (40.50)	139 (49.82)	**0.012**	0.270	0.047	**0.003**
**Living in the Wuhan area, n (%)**	18 (2.44)	59 (3.30)	19 (6.81)	**0.003**	0.256	**0.001**	**0.004**
**Having symptoms or a diagnosis of** **COVID-19, n(%)**	41 (5.56)	75 (4.19)	35 (12.54)	**< 0.001**	0.134	**< 0.001**	**< 0.001**
**Having relatives or acquaintances contracting COVID-19, n (%)**	90 (12.31)	202 (11.28)	66 (23.66)	**< 0.001**	0.508	**< 0.001**	**< 0.001**
**Getting support from family members, n (%)**				**0.027**	0.407	0.071	**0.007**
Unchanged or more	690 (93.62)	1691 (94.47)	252 (90.32)				
Less	47 (6.38)	99 (5.53)	27 (9.68)				
**Getting support from the outside world, n (%)**				0.258			
Unchanged or more	635 (86.16)	1514 (84.58)	229 (82.08)				
Less	102 (13.84)	276 (15.42)	50 (17.92)				
**ISI score**	6.99 ± 5.48	8.25 ± 5.66	8.42 ± 5.93	**< 0.001**	**< 0.001**	**< 0.001**	1.000
**PHQ9 score**	6.18 ± 4.84	7.00 ± 4.88	7.28 ± 5.27	**< 0.001**	**< 0.001**	**< 0.001**	1.000
**ISER score**	18.79 ± 14.83	21.32 ± 14.57	22.36 ± 16.25	**< 0.001**	**< 0.001**	**< 0.001**	1.000

A total of 258 of the 2806 participants (9.19%) experienced improvements in headache intensity during the COVID-19 pandemic, while 2451 (87.35%) reported no change, and 97 (3.46%) reported aggravation of their headache intensity. The aggravation group had a significantly higher proportion of participants who had relatives or acquaintances who had contracted COVID-19 than the remission group (*P*<0.001) and the unchanged group (*P*<0.001). The aggravation group also had a significantly higher proportion of smokers (*P*<0.001) than the remission group. The aggravation group had significantly higher proportions of participants who lived in the Wuhan area (*P* = 0.006) and received less support from family members during social isolation (*P* = 0.001) and a lower ISI score (*P* = 0.014) than the unchanged group (Table 5).

**Table 5:** Comparison of demographic characteristics among the remission group, unchanged group and aggravation group according to headache intensity

characteristics	The remission group^**1**^ (*n* = 258)	The unchanged group^**2**^ (*n* = 2451)	The aggravation group^**3**^ (*n* = 97)	*P* value	*P* value Post hoc		
					1 vs 2	1 vs 3	2 vs 3
**Male, n (%)**	101 (39.15)	901 (36.76)	41 (42.27)	0.430			
**Age, n (%)**				**0.047**			
< 30	77 (29.84)	563 (22.97)	30 (30.93)				
30–50	163 (63.18)	1731 (70.62)	60 (61.86)				
> 50	18 (6.98)	157 (6.41)	7 (7.21)				
**Education level, n (%)**				**0.019**	**0.005**	0.209	0.825
junior colleague or lower	70 (27.13)	483 (19.71)	20 (20.62)				
University or above	188 (72.87)	1968 (80.29)	77 (79.38)				
**Chronic disease history, n (%)**	68 (26.36)	567 (23.13)	28 (28.87)	0.238			
**Smoking, n (%)**	53 (20.54)	398 (16.24)	21 (21.65)	**< 0.001**	**< 0.001**	**< 0.001**	0.083
**Drinking, n (%)**	121 (46.90)	1010 (41.21)	49 (50.52)	**0.048**	0.078	0.543	0.068
**Living in the Wuhan area, n (%)**	11 (4.26)	77 (3.14)	8 (8.25)	**0.019**	0.334	0.137	**0.006**
**Having symptoms or a diagnosis of** **COVID-19, n(%)**	20 (7.75)	121 (4.94)	10 (10.31)	**0.015**	0.053	0.440	**0.030**
**Having relatives or acquaintances contracting COVID-19, n (%)**	47 (18.22)	276 (11.26)	35 (36.08)	**< 0.001**	**< 0.001**	**< 0.001**	**< 0.001**
**Getting support from family members, n (%)**				**0.002**	0.060	0.170	**0.001**
Unchanged or more	236 (91.47)	2313 (94.37)	84 (86.60)				
Less	22 (8.53)	138 (5.63)	13 (13.40)				
**Getting support from the outside world, n (%)**				0.112			
Unchanged or more	221 (85.66)	2082 (84.94)	75 (77.32)				
Less	37 (14.34)	369 (15.06)	22 (22.68)				
**ISI score**	7.52 ± 5.57	8.04 ± 5.68	6.50 ± 5.44	**0.009**	0.494	0.225	**0.014**
**PHQ9 score**	6.83 ± 5.34	6.85 ± 4.90	5.80 ± 4.38	**0.110**			
**ISER score**	20.82 ± 15.42	20.86 ± 14.85	18.03 ± 13.39	0.294			

**Fig. 4:**
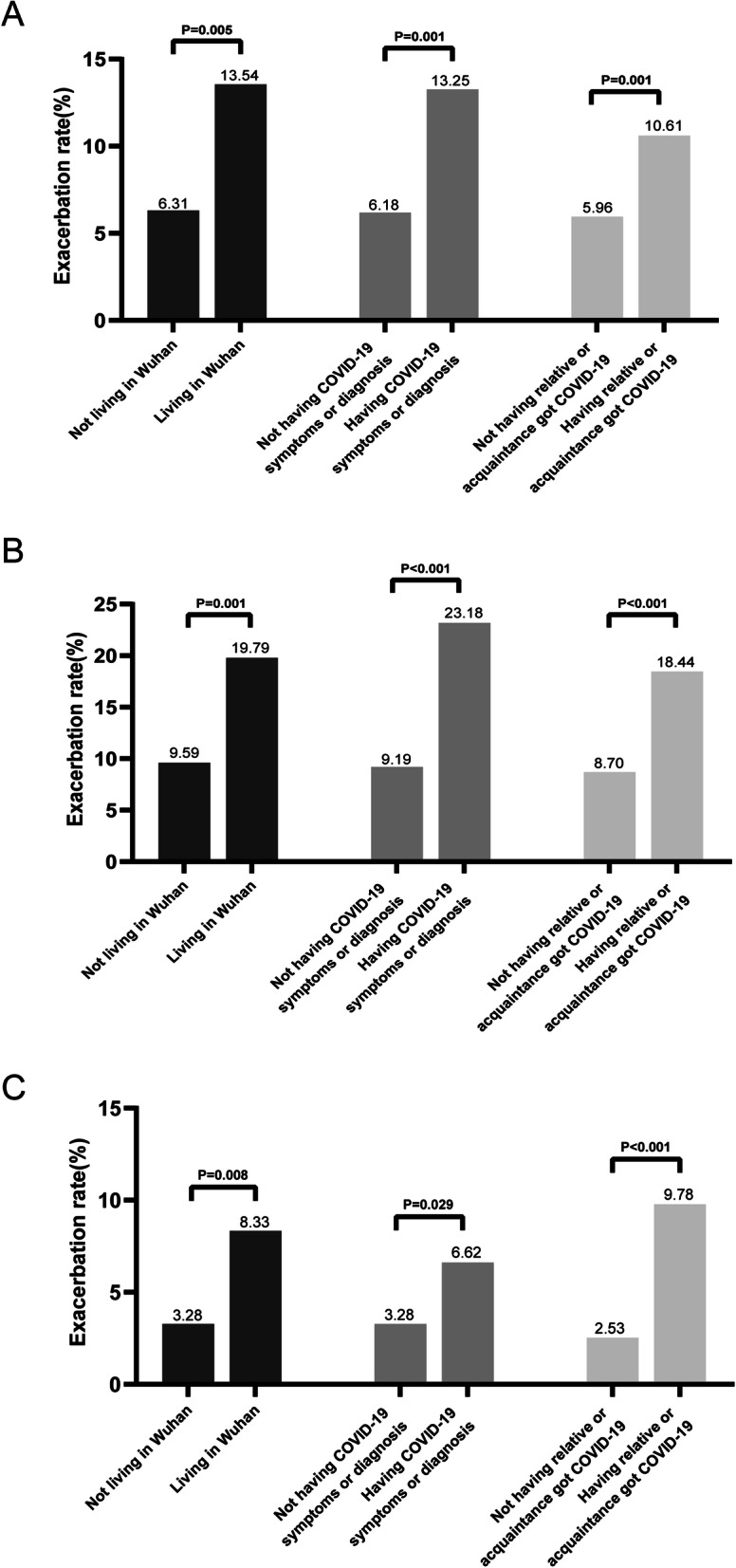
Factors exacerbating headache duration (**A**), headache frequency (**B**) and headache intensity (**C**)

Participants living in the Wuhan area, having COVID-19 symptoms or a diagnosis of COVID-19 and having a relative or acquaintance who had contracted COVID-19 were more likely to experience the exacerbation of headache duration (13.54% vs 6.31%, *P* = 0.005; 13.25% vs 6.18%, *P* = 0.001; 10.61% vs 5.96%, *P* = 0.001), number of headache days (19.79% vs 9.59%, *P* = 0.001; 23.18% vs 9.19%, *P*<0.001; 18.44% vs 8.70%, *P*<0.001) and headache intensity (8.33% vs 3.28%, *P* = 0.008; 6.62% vs 3.28%, *P* = 0.029; 9.78% vs 2.53%, *P*<0.001) (Fig. 4).

### Correlations

Details of the multivariate logistic regression analyses are shown in Tables 6, 7 and 8. Smoking (OR = 1.397, 95% CI 1.090 to 1.790, *P* = 0.008) and receiving support from family members during social isolation (OR = 1.656, 95% CI 1.075 to 2.550, *P* = 0.022) were independent factors affecting the remission of headache duration. Education level (OR = 1.478, 95% CI 1.103 to 1.980, *P* = 0.009) and having a relative or acquaintance who contracted COVID-19 (OR = 0.643, 95% CI 0.458 to 0.902, *P* = 0.011) were independent factors affecting the remission of headache severity.

**Table 6:** Predictors of the remission of headache duration

Variables	EXP(B) (95% CI)	*P* value
Smoking^a^	1.397 (1.090–1.790)	**0.008**
Getting support from family members^b^	1.656 (1.075–2.550)	**0.022**
Getting support from the outside world^c^	1.218 (0.938–1.580)	0.139
ISI score	1.013 (0.992–1.034)	0.232
PHQ9 score	1.006 (0.982–1.031)	0.619
ISER score	1.009 (1.002–1.016)	**0.016**

^a^non-smoking vs smoking

^b^Getting Unchanged or more support from family members vs Getting less support from family members

^c^Getting Unchanged or more support from the outside world vs Getting less support from family members

**Table 7:** Predictors of the remission of headache frequency

Variables	EXP(B) (95% CI)	*P* value
ISI score	0.968 (0.948–0.989)	**0.003**
PHQ9 score	0.997 (0.973–1.022)	0.809
ISER score	0.994 (0.987–1.002)	0.131

**Table 8:** Predictors of the remission of headache intensity

Variables	OR (95% CI)	*P* value
Having relatives or acquaintances contracting COVID-19^a^	0.643 (0.458–0.902)	**0.011**
Education level^b^	1.478 (1.103–1.980)	**0.009**

^a^Not having relative or acquaintance got COVID-19 vs having relative or acquaintance got COVID-19

^b^junior colleague or lower vs University or above

## Discussion

To our knowledge, the present study is the first to investigate the impact of the COVID-19 pandemic on headache patients in China. Our analysis showed reductions in the duration of headache, number of headache days per month, and headache intensity during the COVID-19 pandemic. Smoking, education level and receiving support from family members during social isolation were independent factors affecting the remission of headaches during the COVID-19 pandemic. In addition, participants living in the Wuhan area, having COVID-19 symptoms or a diagnosis of COVID-19 and having relatives or acquaintances with COVID-19 were more likely to experience the worsening of their headaches.

The prevalence of pre-existing headache disorders in the present study was 18.71%, which was slightly lower than that in a previous study^11^. Similar to a previous study [10], our analysis showed a higher prevalence of headache disorders in females (20.57%) than in males (16.22%). Additionally, the prevalence of headache in children and adolescents has been shown to be high and is a major factor associated with school absenteeism and a worse quality of life [11, 12]. Headache increases the risk of physical or psychological problems not only in childhood but also in adulthood [13]. Therefore, it is vital for us to understand the clinical features and evolution of headache from childhood to adulthood.

A previous study showed that migraine patients from the Italian National Headache Registry showed a mild improvement in migraine characteristics during the COVID-19 pandemic [14]. The main aim of this research was to investigate the impact of the COVID-19 pandemic on headache patients in China. Interestingly, but not surprisingly, our analysis showed that headache participants tended to experience improvements in headache duration, headache frequency and headache intensity during the COVID-19 outbreak. We inquired about headache patients and our friends and colleagues. In line with the present study, the respondents reported a tendency towards improvements in headache attacks during social isolation. We also observed that those with migraines were more likely to experience the remission of headache duration than those with TTH and CH. Further work is needed to determine the underlying causes.

Obtaining support from family members during social isolation was an important factor affecting the remission of headache duration. A previous study demonstrated that patients from functional families appeared to have relatively low levels of distress, regardless of the severity of their headache [15]. The COVID-19 outbreak began when the Spring Festival was approaching, which is a period during which family members reunite in China. The Spring Festival period was extended due to the COVID-19 outbreak. Families spent time together during this period and had more time to communicate with and listen to each other. Similarly, participants with pre-existing headache disorders had more opportunities to communicate with family members about their headache-related problems and obtained more effective support, thus reducing their headache-related mental burden. Therefore, we call for more family support to help headache patients improve or eliminate their headache attacks. In addition, time off is important for workers or students to recover from the cumulative effects of work or study [16]. As suggested in a previous study [17], social isolation gave us the opportunity to spend more time at home while avoiding stressful social interactions, which might be another possible reason for the improvements in headaches. Participants with a junior college degree or less education were more likely to experience a remission in headache severity during the COVID-19 pandemic than the participants with a university degree. College students, especially graduate students, experience relatively more pressure from heavy work loads and the need to seek employment. Due to the COVID-19 pandemic, they were not allowed to return to school to conduct research. Given the pressure imposed by the COVID-19 pandemic and academic demands, they have a higher risk of developing many psychological problems, which then result in an aggravation of pre-existing headache disorders. This further suggests that a reduction in stress from work or academics might be another important factor related to headache remission during social isolation. In addition, sleep quality is an acknowledged factor affecting headache attacks. Our analysis showed that the ISI scores were significantly lower in the remission group than in the aggravation group. This result indicated that participants had better sleep quality during the extended holiday, which might be one of the reasons for the relief from headaches that they experienced. In addition, healthier diet habits, such as eating regularly during the extended holiday, might also have played a role. Previous studies reported that not eating on time was a factor precipitating headache attacks [18, 19]. The underlying mechanism could be explained by the development of hypoglycaemia, which activates the adrenal medulla [16]. In particular, smoking was found to be a factor precipitating headache attacks. Participants who did not smoke were more likely to experience the remission of headache duration.

Although there was an overall trend towards the remission of headache, the adverse impacts of COVID-19-related events on pre-existing headache disorders should not be overlooked. Participants who lived in the Wuhan area, had COVID-19 symptoms or a diagnosis of COVID-19 and had relatives or acquaintances who contracted COVID-19 were more likely to experience a deterioration in their pre-existing headache disorder. The COVID-19 outbreak is a stressful event, causing varying degrees of anxiety and worry, and some individuals may even develop post-traumatic stress disorder. Headache participants are more vulnerable to the adverse effects of stressful events. Therefore, it is understandable that COVID-19-related events were factors precipitating headache attacks. Most of the participants included in the present study lived outside of Wuhan, did not have COVID-19 symptoms or a diagnosis of COVID-19 diagnosis and had no relatives or acquaintances who contracted COVID-19. Most people were relatively less affected by the COVID-19 outbreak and thus tended to experience no changes or improvements in their headaches.

A previous study [20] reported that the rate of seropositivity for antibodies (IgM or IgG) against SARS-CoV-2 was 0.8% in the general Chinese population. In the present study, the overall prevalence of COVID-19 was 0.15%, which roughly matched the prevalence of COVID-19 in the Chinese population. Given the large sample size, the results reported in the present study are likely generalizable. However, we acknowledge some limitations of our study. First, since the study was based on a self-administered questionnaire, it was inevitably affected by recall bias. In addition, it was difficult for us to identify which types of headache the respondents had. Additionally, these diagnoses were only based on the information provided in the survey, and secondary causes of headache could not be excluded. Further work is needed. Second, the number of participants who contracted COVID-19 in our study was very small. We could not determine impact of COVID-19 on headache attacks. The prevalence of SARS-CoV-2 infections in headache participants was 0.2% (6/2806). No significant difference was found in the prevalence of SARS-CoV-2 infections between participants with and without pre-existing headache disorders (16/12194, 0.13%). Additionally, headache participants who had confirmed cases of COVID-19 had a higher rate of headache exacerbation than those who did not have COVID-19 (50.00% vs 3.36%, *P* = 0.001). Further study is needed to determine the impact of COVID-19 on headache attacks. Third, sampling error was unavoidable given the use of the online questionnaire; for example, illiterate individuals, elderly individuals, children and those who cannot afford to buy a smartphone may have been less well represented in this study. Fourth, some important information was not obtained with our questionnaire, including some important triggers, such as wearing a mask; the response to analgesics; important accompanying symptoms, such as anosmia; BMI; and marital status. In addition, other factors that might be associated with better headache outcomes were not investigated, such as the regularity of food intake, participation in sports activities, and work-related stress.

## Conclusion

The present study provides the first data about the impact of the COVID-19 pandemic on headache patients in China. The adverse impacts of COVID-19-related events on headache patients, especially those living in high-risk areas, should not be overlooked, although there was an overall trend towards headache remission during the COVID-19 pandemic. Smoking, education level and support from family members during social isolation were important factors associated with better headache outcomes. We call for more family support to help headache patients improve or eliminate headache attacks.

## Supplementary Information

**Additional file 1.** Basic demographic features of all headache participants.

## Data Availability

The datasets are available from the corresponding author on reasonable request.

## Abbreviations

COVID-19: Coronavirus disease 2019
PHQ-9: Patient Health Questionnaire-9
ISI: Insomnia Severity Index
IES-R: Impact of Event Scale Revised
HIS: International Headache Society
DSM-IV: Diagnostic and Statistical Manual of Mental Disorders, Fourth Edition
TTH: Tension-type headache
CH: Cluster headache
