# Supplementary figures and images for: Overall trend towards headache remission during the COVID-19 pandemic among Chinese patients with pre-existing headache highlights the role of family support
Source: BMC Neurol. 2021 Jun 15;21:224. doi: 10.1186/s12883-021-02216-6 (PMC8203488; doi:10.1186/s12883-021-02216-6)

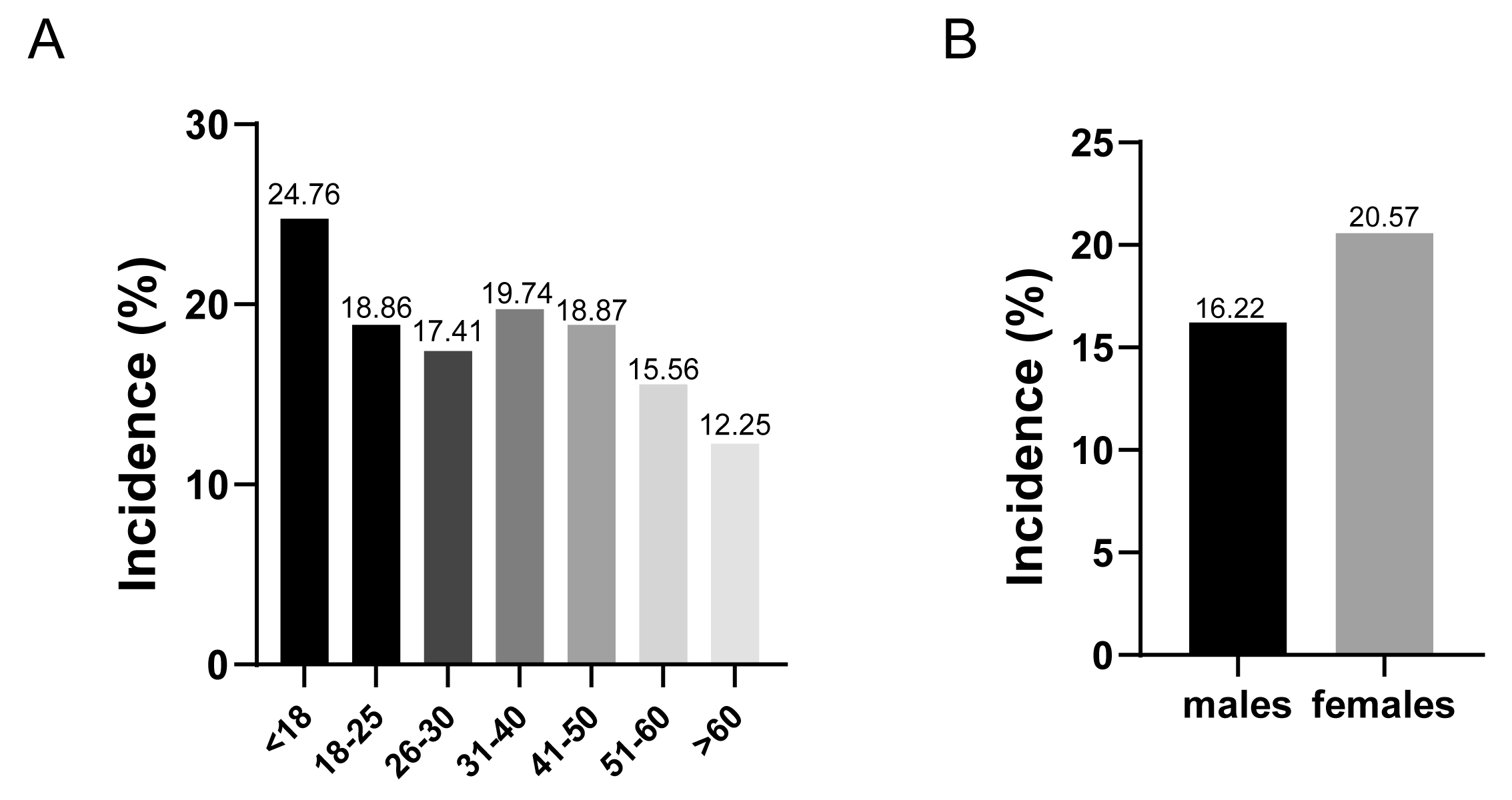

Supplement: Supplementary file 1 — Additional file 1. Basic demographic features of all headache participants. [file 12883_2021_2216_MOESM1_ESM.tif]
